# Social determinants of health in the Mixtec and Zapotec community in Ventura County, California

**DOI:** 10.1186/s12939-015-0148-0

**Published:** 2015-02-03

**Authors:** Annette E Maxwell, Sandra Young, Catherine M Crespi, Roena Rabelo Vega, Reggie T Cayetano, Roshan Bastani

**Affiliations:** Fielding School of Public Health and Jonsson Comprehensive Cancer Center, UCLA Kaiser Permanente Center for Health Equity, University of California, 650 Charles Young Drive South, A2-125 CHS, Box 956900, Los Angeles, CA 90095-6900 USA; Mixteco/Indigena Community Organizing Project, PO Box 20543, Oxnard, CA 93034 USA

**Keywords:** Household survey, Indigenous farm workers, Community-engaged research, Promotoras, Social determinants of health

## Abstract

**Introduction:**

There are an estimated 165,000 indigenous Mexicans living in California, including Mixtec and Zapotec immigrant farm workers. Because many of these immigrants speak only their native non-written languages, there is little information about the needs of this community. An academic-community partnership research team developed a survey to assess basic needs that are known to be social determinants of health in the Mixtec and Zapotec community in Ventura County.

**Methods:**

In summer 2013, Spanish-Mixteco and Spanish-Zapoteco bilingual promotoras conducted surveys in Spanish, Mixteco and Zapoteco in the greater Oxnard area in Ventura County, California to assess the following basic needs: ability of adults and children to obtain health services; household needs regarding work opportunities, food, housing, transportation, safety and education; and discrimination. Independent variables included respondent characteristics such as age, gender, marital status, living part of the year in another city, and household characteristics such as Spanish spoken in the household, number of household members and number of health care providers/agencies used. Several sets of analyses examined the relationship between basic needs and independent variables.

**Results:**

Respondents (N = 989) reported insufficient employment opportunities (74%), food for the family (59%) or housing (48%), lack of transportation (59%), and discrimination or bullying (34%). Most reported access to medical care for children (90%), but only 57% of respondents were able to get health care for themselves.

**Conclusions:**

Many basic needs in the Mixtec and Zapotec community in Ventura County are unmet. It will require many different resources and services to address the needs of this community and to overcome longstanding inequities that are experienced by immigrant farm workers. Our findings will guide the development of future health programs and will serve as a baseline to evaluate the impact of services to improve the health conditions in this community.

## Introduction

There are an estimated 165,000 indigenous Mexicans living in California [1]. Many are farm workers, a group that has historically been exploited as a cheap source of labor [2]. Few studies have focused on indigenous communities in Mexico [3-6] or in the United States [7-11].

Mixtecs and Zapotecs, two indigenous groups from the Oaxaca area in Mexico, have settled in large numbers in Ventura County, California, and many are employed as farm workers with little pay and only seasonal work. Research in these and other immigrant communities is challenging due to language barriers, lack of trust, long working hours, and fear of deportation among some members of the community [7]. While some Mixtecs and Zapotecs speak Spanish and/or English, others speak only their native languages, Mixteco or Zapoteco, which are oral, not written languages. This poses additional challenges for survey research. Consequently, data on the demographic characteristics of this community and its needs are lacking.

The Mixteco/Indigena Community Organizing Project (MICOP), a nonprofit organization, and the University of California Los Angeles Fielding School of Public Health partnered to conduct a community assessment that would provide local data regarding the needs of the Mixtec and Zapotec community. Both partners planned the study, applied for funding, agreed on scope of work and budgets, and worked closely together throughout the whole study, including data analysis and interpretation. Following principles of community-based participatory research [12,13], the partners decided to focus on assessing basic needs that are known to be social determinants of health, including employment, food security, housing, access to health care, transportation, safety and discrimination. The health impact pyramid framework postulates that addressing these basic needs has the greatest potential to improve health [14]. The purpose of this paper is to describe the results of community-based participatory research in which indigenous promotoras conducted a large-scale needs assessment.

## Methods

### Development of survey instrument in English, Spanish and indigenous languages

MICOP, UCLA and a Mixtec Advisory Board worked together to develop a survey that assessed the following basic needs: ability of adults and children to obtain health services; household needs regarding work opportunities, food, housing, transportation, safety and education; and discrimination. Because of the need to keep the survey short and simple, these constructs were not further explained. Participants were simply asked if they or someone in their household had experienced problems in these areas and responses were recorded mostly using simple yes/no checklists. We also assessed demographic information on respondents and household information including composition and use of local agencies and health service providers.

The first English language draft of the survey was translated into Spanish for discussion with non-English speaking promotoras and other community members. Modifications were made parallel in the English and the Spanish versions to simplify and clarify questions. After the Spanish language survey was finalized, a small group of bilingual Mixteco and Spanish speaking promotoras audio-recorded a Mixteco version of the survey, which was provided to all promotoras for practicing, to ensure consistency in the administration in the Mixteco language. This standardization was not necessary for the Zapoteco version, because a single promotora administered all Zapoteco surveys.

### Training of promotoras

MICOP identified 8 Mixteco/Spanish and one Zapoteco/Spanish speaking promotoras, who attended a 6 hour training session in Spanish. The training agenda included purpose of the study, principles of research including voluntary participation and confidentiality, recruitment of participants, how to obtain informed consent, the interviewing process and interview role playing. At a follow-up training/debriefing, promotoras stated that almost all of the people they asked were willing to participate; only a total of 10–12 people they approached refused to be interviewed. Reason for refusal was not collected. Promotoras received a stipend for attending the training and for conducting interviews.

### Administration of survey instrument

Working in pairs, promotoras conducted community survey in Spanish, Mixteco and Zapoteco in Oxnard, Port Hueneme and El Rio between May and September 2013. Promotoras explained that this survey was done to learn about health needs in the Mixtec and Zapotec community and obtained oral consent prior to administering the survey. They conducted the majority of surveys going door to door (37%), at parks (53%), and at a school (5%), during the week (86%) and on weekends (14%), and noted responses on the Spanish language surveys. Surveys were conducted anonymously on the advice of the Mixtec Advisory Board that feared that many community members would not participate if they had to give their name. The budget for this study did not allow payment of participants. Instead and based on community suggestions, participants received an accordion plastic folder for document storage as a token of appreciation. All project activities were approved by the Institutional Review Board of the University of California, Los Angeles.

### Measures

Seven of the basic needs items were asked as yes/no questions, e.g., “Is your housing situation sufficient for your needs?” The following three items had 3 possible responses: “Are you able to get to the places you need to go?” – yes/no/only to some places; “Are you able to work enough of the year to support your family?” – yes/no/yes, but I am struggling; “Does your family have enough food to eat?“ – yes/no/sometimes. All 10 items were dichotomized as basic need met (“yes” response) versus not met (other response). Two questions on discrimination were reverse coded, e.g., “Have you experienced discrimination or bullying?”

Respondents were also asked if they had used the services of 12 local agencies and health service providers and were shown the logos of these providers to aid in recall.

### Analysis

Descriptive statistics are provided for respondent characteristics. Associations among the 10 basic needs items were assessed using Yule’s Q (equivalent to the gamma statistic for two binary variables) and chi-square tests. Guidelines for interpreting the absolute value of Yule’s Q are: 0 to .24, virtually no relationship; .25 to .49, weak relationship; .50 to .74, moderate relationship; .75 to 1.0, strong relationship [15]. Several sets of analyses examined the relationship between basic needs and independent variables. The independent variables included gender, marital status (married/living together versus other), living part of the year in another city (yes/no), Spanish spoken in the household (yes/no), age of respondent (<30 years versus ≥30 years), number of household members (<5 versus ≥5) and number of health care providers/agencies used (>6 versus ≤6). The last three variables were dichotomized at their medians. Chi-square tests were used to examine the bivariate relationship between each basic need and each independent variable. In addition, for each respondent, we computed a total needs met score by summing the number of basic needs met, and used this score as the dependent variable in t tests. Each of the 10 basic needs measures were also used as dependent variables in multivariate logistic regression models including all independent variables.

## Results

A total of 989 respondents completed the survey. As shown in Table 1, respondents were predominantly female, married or living together and all were born in Mexico. The average age of respondents was 31 years. Household size averaged 4.9 members. Languages spoken at home included Spanish and Mixteco (54%), Mixteco only (23%), Spanish and Zapoteco (8%), Spanish only (8%), and English and at least one other language (7%).

**Table 1:** **Demographic characteristics of survey respondents (N = 989), Ventura County, California, 2013**

**Characteristics**	N*		**%**
Female	834/988		84
Married/living as married	847/970		87
Has lived at same address for 12 months	791/984		80
Spent part of the year in another city	144/961		15
Place of birth			
Oaxaca	818/966		85
Guerrero/other	148/966		15
Has at least one child living in Mexico	294/969		30
Languages spoken at home			
Spanish & Mixteco	524/972		54
Mixteco only	224/972		23
Spanish & Zapotec	79/972		8
Spanish only	77/972		8
English & another language	64/972		7
Other language combinations	4/972		0.4
Heard of MICOP prior to survey	726/963		75
	**Mean (Range)**	**Median**	**SD**
Age of respondent	31.2 (18 – 86)	30	9.4
Number of adults in household	2.2 (1 – 10)	2	0.9
Number of children in household	2.7 (0 – 10)	3	1.6
Total number of household members	4.9 (1 – 13)	5	1.9
Number of healthcare agencies/services used	6.0 (0 – 12)	6	2.5

*Denominators vary due to missing responses.

MICOP = Mixteco/Indigena Community Organizing Project.

**Figure 1:**
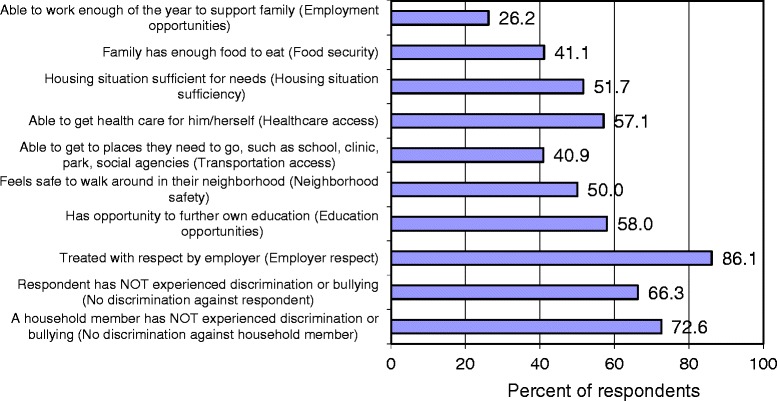
**Coverage of basic needs among respondents.**

Figure 1 shows overall levels of basic needs. Only 26% of respondents reported that they were able to work enough of the year to support their family and 41% reported that their family had enough food. Only 52% of respondents reported sufficient housing, 57% reported having access to health care, 41% reported access to transportation, 50% reported neighborhood safety and 58% reported education opportunities. Eighty-six percent of respondents stated that they were treated with respect by employers, but 34% had experienced discrimination or bullying and 27% reported that another household member had experienced discrimination or bullying. Twenty five percent of the sample had only 3 basic needs met out of the 10 that were assessed and 22% of the sample had 8 or more needs met. Most households included children (90%, data not shown) and the majority of respondents of those households reported access to medical care for children (90%), being able to support children’s success in school (89%) and having childcare/early education opportunities available for their family (76%).

Table 2 shows moderate (Q = .50 to .74) to strong (Q ≥ .75) relationships among employment opportunities, food security, housing condition sufficiency, healthcare access and transportation access. Weaker relationships are found among indicators of neighborhood safety, employer respect and discrimination, and between these measures and the first group of variables. Education opportunities showed little relationship with the other measures.

**Table 2:** **Associations among basic needs in the Mixtec/Zapotec community in Ventura County, California, 2013 (N = 989)**

**Basic needs items**	**Yule’s Q**									
	**Employment opportunities**	**Food security**	**Housing condition sufficiency**	**Healthcare access**	**Transportation access**	**Neighborhood safety**	**Education opportunities**	**Employer respect**	**No discrimination of respondent**	**No discrimination of household member/s**
Employment opportunities	1	.82*	.60*	.68*	.82*	.55*	.12	.48*	.42*	.37*
Food security		1	.66*	.65*	.78*	.39*	-.02	.34*	.30*	.39*
Housing condition sufficiency			1	.69*	.60*	.43*	-.06	.32*	.39*	.35*
Healthcare access				1	.72*	.53*	-.19	.19	.28*	.39*
Transportation access					1	.41*	.01	.40*	.31*	.37*
Neighborhood safety						1	.06	.48*	.31*	.28*
Education opportunities							1	.24	-.11	-.05
Employer respect								1	.53*	.53*
No discrimination of respondent									1	.93*
No discrimination of household member/s										1

Asterisk (*) indicates association is statistically significant at .001 level using chi-square test.

Table 3 reports the results investigating how respondent characteristics are associated with basic needs. The total number of basic needs met was higher among male respondents than among females; differences in employment and education opportunities and transportation access accounted for much of this difference. Married respondents reported more needs met than single respondents, with greater healthcare access and employment opportunities contributing to the higher scores. Respondents under 30 years of age reported more needs met than older respondents for most (7 out of 10) basic needs. Similarly, smaller households reported significantly more needs met than larger households, with higher rates of needs met for food security, housing sufficiency, healthcare access, and freedom from discrimination. Respondents who had used more than 6 health and social services also reported more needs met. Respondents who had spent time in another city reported better healthcare access, while those who spoke Spanish reported lower housing sufficiency and healthcare access but better education opportunities and employer respect.

**Table 3:** **Summary of basic needs met by characteristics of respondents (N = 978), Ventura County, California, 2013**

	**Proportions reporting basic need met**										
	**Employment opportunities**	**Food security**	**Housing situation sufficiency**	**Healthcare access**	**Transportation access**	**Neighborhood safety**	**Education opportunities**	**Employer respect**	**No discrimination against respondent**	**No discrimination against household member/s**	**Mean total number of needs met (± SD)**
Male respondent (n = 154)	.39***	.49*	.53	.58	.55***	.43	.66**	.82	.73*	.70	5.8 (±2.6)*
Female respondent (n = 834)	.24	.40	.51	.51	.38	.50	.53	.83	.64	.67	5.3 (±2.5)
Married/living as (n = 847)	.27**	.42	.52	.59***	.42*	.50	.54*	.84*	.65	.68	5.4 (±2.5)*
Single (n = 123)	.16	.34	.46	.40	.30	.42	.66	.76	.69	.66	4.8 (±2.3)
Spent time in another city (n =144)	.29	.43	.55	.67**	.47	.50	.52	.83	.72	.67	5.7 (±2.6)
Did not (n = 817)	.26	.41	.51	.55	.40	.49	.56	.82	.64	.68	5.3 (±2.5)
Speaks Spanish (n = 745)	.27	.40	.47***	.51***	.40	.50	.61***	.84*	.66	.68	5.4 (±2.5)
Does not speak Spanish (n = 227)	.22	.44	.65	.74	.42	.46	.36	.77	.64	.66	5.4 (±2.4)
Age < 30 years (n = 473)	.30**	.46**	.57***	.60*	.44*	.53*	.58	.83	.71**	.71	5.7 (±2.4)***
Age ≥ 30 years (n = 505)	.22	.37	.45	.52	.37	.46	.53	.83	.61	.65	5.0 (±2.5)
Household members < 5 (n = 436)	.28	.47**	.59***	.62**	.44	.51	.56	.82	.71**	.73**	5.7 (±2.5)***
Household members ≥ 5 (n = 492)	.24	.37	.44	.53	.38	.47	.55	.83	.62	.64	5.1 (±2.4)
Used > 6 services (n = 490)	.31**	.45*	.51	.61**	.46**	.48	.53	.83	.65	.69	5.5 (±2.6)*
Used ≤ 6 services (n = 499)	.22	.37	.51	.52	.35	.50	.57	.82	.66	.66	5.2 (±2.4)

*significant at p ≤ .05; **significant at p ≤ .01; ***significant at p ≤ .001 (chi-square tests for proportions and t tests comparing mean total number of needs met).

Table 4 presents adjusted odds ratios for the association of each predictor with each basic needs measure. Associations were similar to those found in the unadjusted analyses, with some notable differences. Males were significantly more likely than females to report sufficient employment opportunities, food security, access to transportation and education opportunities. Respondents who were married or living as married were more likely than single respondents to report basic needs covered for five of the ten needs assessed. Respondents from Spanish speaking households were more likely to report sufficient education opportunities and being treated with respect by employers than respondents from non-Spanish speaking households, but they were less likely to report sufficient housing and having access to healthcare for themselves. When adjusting for other characteristics, age was no longer a significant factor for most needs; only sufficient housing and no discrimination were met with higher odds than for older respondents. Respondents from smaller households (less than 5 household members) were more likely to report food security, sufficient housing and access to healthcare and transportation than respondents from larger households. Respondents who had used more than 6 out of 12 local agencies and/or health service providers were more likely to report sufficient employment opportunities, food security and access to healthcare and transportation than respondents who had used fewer service providers.

**Table 4:** **Adjusted odds ratios from multivariate logistic regression models predicting basic need items among respondents (N = 978), Ventura County, California, 2013**

	**Outcomes**									
	**Employment opportunities**	**Food security**	**Housing situation sufficiency**	**Healthcare access**	**Transportation access**	**Neighborhood safety**	**Education opportunities**	**Employer respect**	**No discrimination against respondent**	**No discrimination against household member/s**
Male (Ref: female)	2.0*** (1.3-3.0)	1.6* (1.1-2.4)	1.1 (0.8-1.7)	0.8 (0.6-1.2)	2.1*** (1.4-3.1)	0.7 (0.5-1.0)	1.6* (1.0-2.3)	0.8 (0.5-1.4)	1.4 (0.9-2.1)	1.1 (0.7-1.6)
Married/living as married (Ref: single)	1.9* (1.1-3.4)	1.5 (0.9-2.3)	1.6* (1.1-2.5)	2.1*** (1.4-3.3)	1.8* (1.1-2.8)	1.5 (1.0-2.2)	0.6* (0.4-0.9)	1.8* (1.1-3.0)	0.7 (0.5-1.2)	1.1 (0.7-1.8)
Spent time in another city (Ref: did not)	1.1 (0.7-1.7)	1.0 (0.7-1.5)	1.0 (0.7-1.5)	1.7* (1.1-2.5)	1.4 (0.9-2.0)	1.0 (0.7-1.4)	0.7 (0.5-1.0)	1.1 (0.6-1.7)	1.4 (0.9-2.2)	1.0 (0.6-1.4)
Speaks Spanish (Ref: does not)	1.1 (0.8-1.7)	0.8 (0.6-1.1)	0.4*** (0.3-0.6)	0.4*** (0.3-0.6)	0.9 (0.7-1.3)	1.2 (0.9-1.7)	2.9*** (2.1-4.0)	1.6* (1.1-2.4)	1.1 (0.8-1.5)	1.1 (0.8-1.5)
Age < 30 years (Ref: ≥ 30 years)	1.4 (1.0-1.9)	1.3 (1.0-1.7)	1.4* (1.0-1.8)	1.3 (0.9-1.7)	1.2 (0.9-1.6)	1.2 (0.9-1.6)	1.3 (1.0-1.7)	0.9 (0.6-1.3)	1.5** (1.1-2.0)	1.2 (0.9-1.6)
Household size < 5 (Ref: ≥ 5)	1.3 (0.9-1.8)	1.6** (1.2-2.1)	1.8*** (1.3-2.4)	1.6** (1.2-2.1)	1.4* (1.1-1.9)	1.2 (0.9-1.6)	1.0 (0.7-1.4)	1.0 (0.7-1.5)	1.3 (0.9-1.7)	1.6** (1.2-2.2)
Used > 6 services (Ref: ≤ 6)	1.8*** (1.3-2.5)	1.5** (1.1-2.0)	1.1 (0.8-1.5)	1.5** (1.2-2.1)	1.8*** (1.3-2.4)	0.9 (0.7-1.2)	1.0 (0.7-1.3)	1.0 (0.7-1.4)	1.1 (0.8-1.4)	1.2 (0.9-1.7)

All odds ratios are adjusted for all other predictors in the model.

*significant at p ≤ .05; ** significant at p ≤ .01; ***significant at p ≤ .001 >.

## Discussion

We conducted one of the largest needs assessments among indigenous people from Mexico who have settled in the U.S. Through a community-based participatory research process that included an established and trusted community organization and indigenous promotoras, we were able to conduct almost 1,000 surveys in two indigenous languages and in Spanish in an immigrant community that is largely undocumented and has low levels of income and education. Our method for consistent administration of a needs assessment in a language that does not have a written form might be useful for conducting assessments in other areas of interest and for other indigenous groups that lack a written language. However, we have to acknowledge that this process can also lead to errors if the questions are not asked exactly as written in Spanish or if the responses are incorrectly recorded in the Spanish language survey.

Confirming findings from surveys conducted among much smaller samples of indigenous farm workers in Oregon [10] and California [16,17], our data, collected in the Mixtec and Zapotec community in Ventura County, California, show that many basic needs such as food security, housing and access to health care are not met. These basic needs, which are human rights and social determinants of health, are the foundation of public health efforts [14]. Our data suggest that the majority of children have access to health care, but this access often comes at the price of waiting at the emergency room for many hours to be seen (personal communication, Sandra Young, 4/27/14).

A farm worker study conducted in Fresno, California that focused on food security found large seasonal differences: only 24% of Mixteco-speaking respondents reported sufficient food in winter but 100% reported sufficient food in summer. The researchers attribute this big difference to the fact that the sample recruited in winter included more women with families and an average of 33 hours of work per week, while almost half the respondents in the summer were unaccompanied men that worked on average 60 hours per week [17]. Our study was conducted during the harvest season, and results might have been quite different had we conducted the survey in a season when there are fewer employment opportunities.

Some of the results were unexpected: Although discrimination is described as an important issue among migrant farm workers [10], the majority of adults in our sample reported that they were treated with respect by employers and that nobody in their household had experienced discrimination or bullying. We found that different demographic characteristics were associated with different needs, sometimes in unexpected directions. For example, we had expected that respondents of Spanish-speaking households would have more of their basic needs covered than respondents of non-Spanish-speaking households. While respondents of Spanish-speaking households were more likely to report being treated with respect by employer and having education opportunities than respondents of non-Spanish speaking households, respondents of non-Spanish speaking households were more likely to report access to health care and sufficient housing than respondents of Spanish-speaking households. One reason for this unexpected finding may be lower expectations among non-Spanish speaking community members regarding health care access and housing, rather than better coverage of health care needs.

Per recommendation of the Community Advisory Board, we did not assess immigration status, income or educational level among participants in our study. Another survey that included 60 Mixtec farm workers in Fresno County, California found that 98% of respondents were undocumented, 90% had an education of 6th grade or less and the mean monthly income per person ranged from $271 in winter to $1,927 in summer [17]. Both our and the Fresno study used similar recruitment methods, and based on personal communications with MICOP staff, our sample most likely also included a substantial proportion of undocumented farm workers. In addition to language and other barriers, being undocumented is a major obstacle to accessing health care and social services [18]. Our finding of moderate to strong correlations of basic needs regarding food security, housing, transportation and access to health care suggest that many respondents have multiple needs that have to be addressed simultaneously; however, undocumented farm workers will be extremely hesitant to utilize health and social services (personal communication, Sandra Young, 4/27/14).

### Limitations

All data are based on self-report. The survey had to be short to minimize respondent burden – therefore, the number of questions was limited and questions focused on expected areas of unmet basic needs; we did not assess assets such as the strong sense of community in these Mixtec and Zapotec groups. The majority of respondents were female and males were underrepresented; although many questions assessed household needs, females could have been more forthcoming than males to report unmet needs, which could explain some of the gender differences we found. We interviewed a convenience sample, although given the reported excellent participation rate, the large number of participants and the recruitment at several different locations in the greater Oxnard area, we believe that the survey provided a good snapshot of the basic needs of indigenous farm workers in Ventura County.

## Conclusions

Our survey documents that the Mixtec and Zapotec community in Ventura County experiences major problems in many basic needs. Having local data will draw attention to this community that has often been described as “invisible” [1,19,20]. MICOP has begun the process of sharing information from this survey with local health care providers. However, it will require many different resources and services to address the many needs of this community and to overcome longstanding inequities that are experienced by immigrant farm workers [2,18]. Our findings will guide future programming of MICOP and will serve as a baseline to evaluate the impact of services to improve the health conditions in this community. Both the community and the academic partners of this collaboration have agreed to continue research in this indigenous farmworker community with a focus on increasing the capacity of MICOP and indigenous promotoras to engage in health promotion.
